# Relationship between cytokines and brain-derived neurotrophic factor (BDNF) in trajectories of cancer-related cognitive impairment

**DOI:** 10.1016/j.cyto.2021.155556

**Published:** 2021-05-10

**Authors:** Ning Yi Yap, Yi Long Toh, Chia Jie Tan, Munjal M. Acharya, Alexandre Chan

**Affiliations:** a Department of Pharmacy, Faculty of Science, National University of Singapore, Singapore; b Department of Radiation Oncology, University of California, Irvine, USA; c Department of Clinical Pharmacy Practice, School of Pharmacy & Pharmaceutical Sciences, University of California, Irvine, USA; d Department of Pharmacy, National Cancer Centre Singapore, Singapore

**Keywords:** BDNF, Breast cancer, Cancer-related cognitive impairment, Cytokines

## Abstract

Cytokines facilitate the peripheral immune and cerebral response, through their ability to modulate the expression of brain derived neurotrophic factor (BDNF). Cytokines and BDNF are implicated in cancer-related cognitive impairment (CRCI), but their relationship has not been clearly defined for this condition. The aim of this study was to evaluate the associations of cytokines and BDNF among early stage breast cancer (ESBC) patients with different CRCI trajectories. This was a multicenter longitudinal study involving 136 ESBC patients. CRCI was assessed using the FACT-Cog (V3) questionnaire. Plasma cytokines and BDNF levels were quantified at three time points throughout chemotherapy. The associations between cytokines and BDNF were analyzed using linear mixed models, with interaction terms for CRCI status. All cytokines analyzed showed inverse associations with BDNF levels. There was a significant interaction between IL-6 and the persistent impairment trajectory, which would impact on BDNF levels (p = 0.026). The inverse associations with BDNF were more pronounced for IFN-γ, IL-1β, IL-4, IL-8, and GM-CSF in patients with persistent CRCI. The coefficient values for IL-2, IL-4, and TNF-α also indicate that there was a greater magnitude of decrease in BDNF level for every unit of cytokine increase in patients with acute and persistent CRCI, compared to patients without CRCI. The differential associations between cytokines and BDNF may be indicative of probable susceptibility to the elevation of cytokines. Further research is required to elucidate the specific associations of cytokines and BDNF, along with their contributions to acute and persistent CRCI.

## 1. Introduction

Cancer-related cognitive impairment (CRCI) is a well-documented sequela of cancer treatments such as chemotherapy and radiotherapy, which can adversely impact long-term quality of life. CRCI affects up to 65% of breast cancer patients during treatment and one third of survivors may suffer from lasting cognitive deficits 1 year following the completion of treatment [12,54]. Distinct CRCI trajectories manifest among patients, with some experiencing acute impairment only during treatment while others may have persistent or delayed onset of impairment post-treatment [38]. The underlying predispositions for certain subsets of patients to develop acute or long term CRCI are still obscure, and a clearer understanding of the factors contributing to CRCI is important to develop an effective clinical recourse.

The etiology of CRCI is multifaceted with the involvement of psychological, biological and genetic determinants. One biological factor of interest is brain-derived neurotrophic factor (BDNF), a neurotrophin which is required for neurogenesis and the maintenance of neuronal plasticity [48]. Dysregulation of BDNF levels, along with the BDNF Val66Met polymorphism (rs6265) have been implicated in neuropsychological disorders such as depression, Alzheimer’s disease, dementia and CRCI [1,14,16,25,39]. However, there is mixed evidence on the relationship between rs6265 and BDNF levels in literature as genetic polymorphism alone may not account for BDNF dysregulation [1,16,56]. BDNF expression can be influenced by epigenetic changes and upstream regulators such as cytokines [26,27]. Similar to BDNF, aberrant cytokines levels are affiliated with the aforementioned spectrum of neuropsychological disorders, including CRCI [10,25,29,32,43,51]. The parallel involvement of cytokines and BDNF in the etiology of these conditions suggests that the impact of inflammation on brain function could be attributed to downstream effects on BDNF levels.

Chemotherapy evokes a systemic immune response, leading to an increase in peripheral inflammatory cytokines which are able to cross the blood-brain barrier (BBB), whereas most chemotherapy drugs are impeded due to the larger molecular size [51]. Cytokines can trigger a cascade of local inflammatory response in the brain, resulting in elevated oxidative stress, a compromised BBB and neuroinflammation [47]. Pro-inflammatory cytokines can also suppress BDNF expression in the brain and this inhibitory effect has been described in a number of in vivo studies [20,46,50]. For instance, induction of peripheral immune response by lipopolysaccharide significantly reduced cerebral BDNF expression of animal models [20,50]. The in vivo evidence suggests that cytokines can act as intermediaries for the communication between the peripheral immune and central nervous system (CNS). However, the relationship between circulating cytokines and BDNF in clinical studies has been contradictory. A study in cancer patients with depression showed a significant negative correlation between circulating IL-6 and BDNF, while a positive association was observed in patients with major depressive disorder [25,43]. In patients diagnosed with dementia, serum TNF-α and IL-1β were not correlated with BDNF levels [57]. These inconsistent outcomes highlight that the associations between cytokines and BDNF have yet to be fully elucidated and varying responses may be seen in different conditions. To date, the link between circulating cytokines and BDNF in patients with CRCI has not been described. An elaborated investigation of this relationship would help characterize the contributions of circulating cytokines in modulating BDNF levels among cancer patients who experience CRCI and facilitate the identification of potential crucial upstream targets aimed at improving BDNF levels and cognitive function.

Previously, we have shown that elevated levels of pro-inflammatory cytokines IL-1β and IL-6 corresponded with poorer cognitive response and higher BDNF levels were protective against persistent CRCI in a cohort of early stage breast cancer (ESBC) patients [10,56]. The aim of the present study was to investigate associations of cytokines and BDNF among ESBC patients with CRCI in comparison to those without CRCI. The differential associations of cytokines and BDNF for self-reported CRCI trajectories (acute, persistent and delayed) were also evaluated.

## 2. Methods

### 2.1. Study participants

This was a prospective, longitudinal, cohort study and study participants were recruited from the National Cancer Center Singapore, Changi General Hospital and KK Women’s and Children’s Hospital. This study was granted ethical approval from the SingHealth Institutional Review Board (CIRB 2014/754/B) with recruitment and follow-ups from November 2014 to January 2020. All participants were consented prior to data collection. Patients who fulfilled the following criteria were recruited for the study: (i) at least 21 years of age, (ii) diagnosis of ESBC (stages I – IIIA), (iii) Have not undergone chemo/radiotherapy and scheduled to receive anthracycline- or taxane-based chemotherapy and (iv) able to read and understand English or Mandarin. Patients were excluded from this study if they were: (i) physically or mentally incapable of providing informed consent or (ii) diagnosed with neuropsychiatric or neurologic medical conditions that would interfere with cognitive functioning.

### 2.2. Study procedures

Participants’ demographic and clinical information was acquired from electronic medical records and participant interviews. Participants were assessed at four time points: before chemotherapy initiation (baseline) (T1), 6 weeks after chemotherapy initiation (T2), 3 months after chemotherapy initiation (at the end of chemotherapy) (T3), and 12–24 months post-chemotherapy completion (T4). At each time point, subjective cognitive function, fatigue, anxiety, and depression were evaluated using patient-reported outcome questionnaires. Blood samples were taken for BDNF genotyping, plasma BDNF and cytokines quantification at T1, T2, and T3.

### 2.3. Patient-reported outcome questionnaires

Subjective cognitive function was assessed using the Functional Assessment of Cancer Therapy-Cognitive Function (FACT-Cog) version 3 questionnaire. The English and Mandarin versions used in this study have been validated in a population of breast cancer patients in Singapore [9]. The questionnaire consists of 37 items and each item is scored on a 5-point Likert-type scale, according to its frequency for the past 7 days. The overall FACT-Cog score was obtained by tabulating all of the individual item scores. Cognitive impairment was defined as having a reduction of at least 10.6 points in the overall FACT-Cog score relative to baseline, in accordance to the previously established minimal clinically important difference (MCID) threshold [7]. Participants were classified into different cognitive trajectories based on their self-reported cognitive function: No clinically significant cognitive impairment at all time points, acute (clinically significant cognitive impairment at T2 and/or T3 but not at T4), persistent (clinically significant cognitive impairment at T3 and T4), and delayed impairment (clinically significant cognitive impairment only at T4) [38].

Anxiety, depressive symptoms and fatigue levels were assessed using the Beck Anxiety Inventory (BAI), Beck Depression Inventory (BDI), and Brief Fatigue Inventory (BFI), and respectively [4,28,33].

### 2.4. Blood sample collection

Whole blood was collected in an ethylene diamine tetraacetic acid (EDTA) tube and centrifuged at 2500 rpm for 10 min. The extracted plasma and buffy coat were stored at −80 °C until analysis.

### 2.5. Plasma cytokines and BDNF quantification

Plasma cytokine levels were determined using the Luminex multiplex panel (Bio-rad, USA) which included granulocyte-macrophage colony-stimulating factor (GM-CSF), interferon gamma (IFN-γ), tumor necrosis factor (TNF-α), interleukin (IL)-1β, IL-2, IL-4, IL-6, IL-8 and IL-10. Plasma cytokine levels were determined from standard curves generated by standards of known cytokine concentrations (Supplementary Figure S1). The concentrations of samples with cytokine levels that were below the lower limit of quantification were substituted with zeros. Cytokines with >80% of values that were below the lower limit of quantification were excluded from the final analysis. Plasma samples were diluted 50-fold for BDNF quantification using a commercially available enzyme-linked immunosorbent assay (ELISA) kit (Biosensis BEK-2211-1P/2P, Australia). Assays were performed in duplicates according to the manufacturer’s instructions and an inter and intra-assay coefficient of variance of less than 15% was considered to be acceptable.

### 2.6. BDNF genotyping

Genomic DNA was extracted from the buffy coat using the QIAmp DNA Blood Mini Kit (Qiagen, Germany). Genotyping of the Val66Met (rs6265) polymorphism was achieved by polymerase chain reaction (PCR) followed by Sanger sequencing using a 3730 xl DNA Analyzer (Applied Biosystems, USA). The primers for PCR were 5′-GGACTCTGGAGAGCGTGAA-3′ (forward) and 5′-CGTGTACAAGTCTGCGTCCT-3′ (reverse).

### 2.7. Statistical analysis

Statistical analyses were conducted with the Stata version 16 software (StataCorp, USA). Demographics, clinical data and the incidences of cognitive impairment were summarized using descriptive statistics. The Friedman test was used to analyze the changes in plasma cytokines and BDNF levels across time points, followed by the Wilcoxon signed-rank test with Bonferroni correction for post hoc pairwise comparisons.

Linear mixed model analyses, with time point adjustments, were carried out to analyze the associations between plasma cytokines and BDNF levels, with cytokines as the independent variables and BDNF as the dependent variable. Analysis was first performed with interaction terms between cytokines and participants grouped by those without reported CRCI for all assessment time points and those with reported CRCI. Analysis was then performed with interaction terms between cytokines and the different cognitive impairment trajectories (acute, persistent, delayed), with the group without reported CRCI acting as reference. Random intercept was included to account for individual-specific effects. Clinically important factors that could affect BDNF levels such as the rs6265 genotype status, age, BMI and depression status were included in the adjusted models [43,44]. The output was presented as beta coefficient values representing point estimates incorporating the main and interaction effects. A p value of less than 0.05 was considered statistically significant.

## 3. Results

### 3.1. Demographics and clinical characteristics

A total of 217 participants were recruited for the study. After excluding participants with incomplete follow-up data or biomarker samples, 136 participants were included in the final analysis. The participants’ baseline characteristics are presented in Table 1. The mean age ± SD of participants was 52.0 ± 8.9 years and the majority of participants had at least a secondary school level education (87.5%), were diagnosed with stage II breast cancer (66.9%) and had undergone anthracycline-based chemotherapy (70.6%). The baseline characteristics of participants who were retained in the final analysis and those who were removed were comparable, except for the ethnicity, type of chemotherapy regimen and radiotherapy received (Supplementary table S1). Among patients included in the analysis, a larger proportion received anthracycline based chemotherapy and radiotherapy. Patient demographic characteristics were analyzed for their associations with BDNF levels in Supplementary Table S2. Throughout the assessment time points, 40.4% of participants who reported CRCI experienced either acute (11.0%), persistent (15.4%), or delayed (14.0%) impairment (Table 1).

### 3.2. Cytokines and BDNF levels across time points

Among the cytokines analyzed, there was an overall significant increase in plasma concentrations of cytokines IFN-γ, IL-4, IL-6, and TNF-α, from the inception to the end of chemotherapy (T1 to T3) (Table 2). IL-10 was not included in the analysis because >80% of the readings were below the limit of quantification. Plasma BDNF levels were reduced over time (p < 0.001), and there was significant reduction in BDNF levels between T1 versus T2 (p < 0.001) and between T1 versus T3 (p < 0.001).

### 3.3. Associations between cytokines and BDNF

**Fig. 1.**
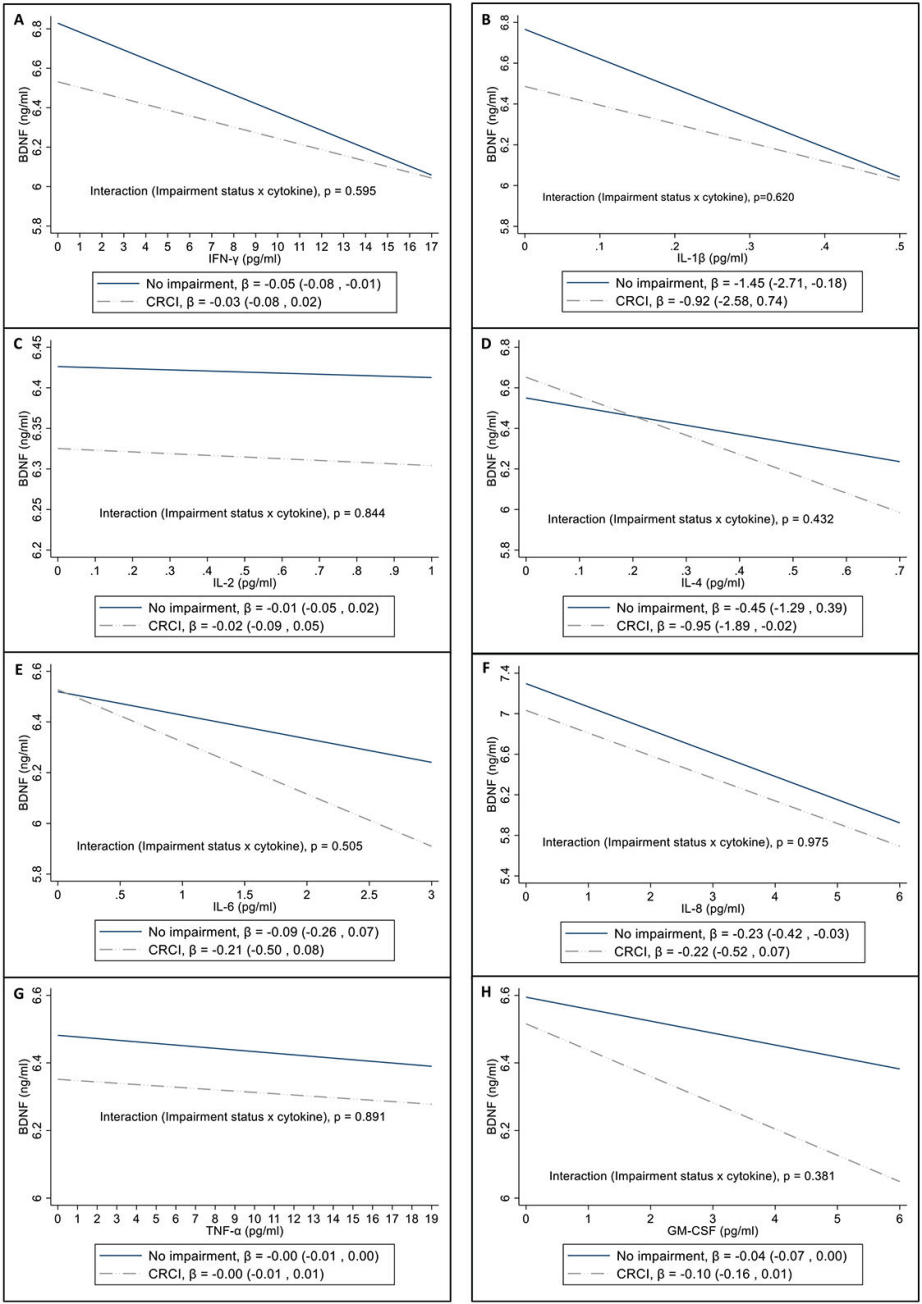
Interaction plots^a^ from linear mixed models investigating the associations of cytokines (pg/ml) and BDNF (ng/ml) in patients with CRCI (pooled) and without CRCI^b^. ^a^ Analysis performed with interaction terms for cytokines and CRCI status (No impairment and CRCI), with the “No impairment” group as reference. Coefficient values represent point estimates incorporating the main and interaction effects. ^b^ Models adjusted for age, BDNF SNP, BMI, depression status and impairment status.

Cytokines were evaluated for their associations with BDNF levels among ESBC patients with and without CRCI (Fig. 1). All cytokines analyzed showed inverse associations with BDNF levels. There were no significant interactions between individual cytokines and cognitive impairment status, when comparing the associations of cytokines and BDNF in patients who reported CRCI during the follow-up period (T2, T3 or T4) and patients who did not experience CRCI.

**Fig. 2.**
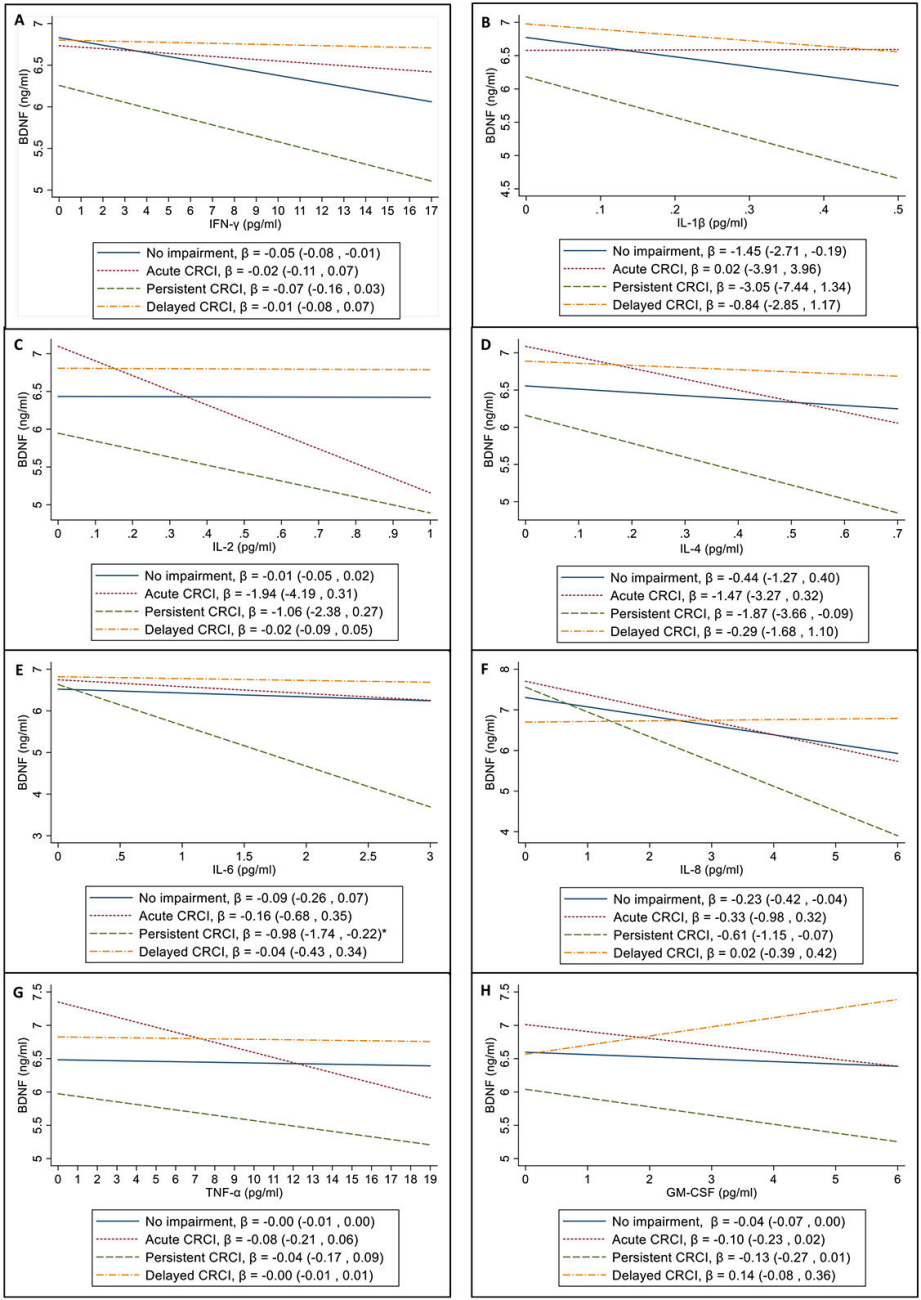
Interaction plots^a^ from linear mixed models investigating the associations of cytokines (pg/ml) and BDNF (ng/ml) in patients with CRCI (acute, persistent, delayed) and without CRCI^b^. ^a^ Analysis performed with interaction terms for cytokines and CRCI trajectory (acute, persistent, delayed), with the “No impairment” group as reference. Coefficient values represent point estimates incorporating the main and interaction effects. ^b^ Models adjusted for age, BDNF SNP, BMI, depression status and impairment status. *1E : Significant interaction between persistent CRCI and IL-6 (p = 0.026).

Due to the heterogenous nature of CRCI, the associations between cytokines and BDNF in patients with different CRCI trajectories (acute, persistent and delayed) were then compared with patients without CRCI, as shown in Fig. 2. A significant interaction for persistent CRCI and IL-6 (p = 0.026) was observed (Fig. 2E). The interactions between other impairment trajectories and other cytokines did not reach statistical significance. However, the coefficient values for indicate that there was a greater magnitude of decrease in BDNF level for every unit of cytokine increase (≥3 fold difference) in patients with acute and persistent CRCI, compared to patients without CRCI (Fig. 2C, 2D & 2G). The negative associations with BDNF were also more pronounced for IFN-γ, IL-1β, IL-4, IL-6, IL-8, and GM-CSF in patients with persistent CRCI, compared to patients exhibiting other cognitive trajectories (no impairment, acute and delayed CRCI). In contrast, the coefficient values were closer to 0 for IFN-γ, IL-2, IL-4, IL-6, IL-8, and TNF-α in patients with delayed CRCI, compared to patients with acute or persistent CRCI (see Fig. 2).

## 4. Discussion

Building upon our previous analyses which demonstrated the protective effects of BDNF against CRCI and the associations of cytokines with cognitive function in ESBC patients, the present study is focused on the modulatory potential of cytokines on BDNF levels [10,53,56]. Specifically, this study is novel because it is the first study to characterize the relationships between cytokines and BDNF in patients with CRCI, in comparison to patients without CRCI. In the current analysis, circulating levels of IFN-γ, IL-1β, IL-2, IL-4, IL-6, IL-8, TNF-α, and GM-CSF exhibited inverse associations with BDNF levels in a cohort of ESBC patients, throughout the course of chemotherapy. These inverse associations indicate a possible inhibitory effect of peripheral cytokines on BDNF levels, which need to be further evaluated in preclinical and in vitro models.

There was an overall trend of circulating cytokine increase, indicative of gross peripheral immune activation by chemotherapy, and a significant decline in BDNF levels throughout chemotherapy. While the mechanism of chemotherapy or cancer-induced inflammation is better defined, the exact cause of chemotherapy related BDNF decline is still unclear. BDNF is mostly secreted by neurons and is found abundantly in various regions of the brain, but is able to cross the BBB contributing to detectable levels in the blood [40]. Although peripheral immune cells such as peripheral blood mononuclear cells (PBMC) and platelets are also sources of BDNF, plasma BDNF levels are found to correlate with and reflect BDNF levels in the brain [6,30,45]. Furthermore, peripheral immune cells may not contribute significantly to peripheral circulating BDNF levels [19]. In this study, the significant downtrend of plasma BDNF levels during chemotherapy implies a major decline in the CNS contribution of circulatory BDNF. Preclinical studies have shown that exposure to cytotoxic agents significantly reduces proliferating neural stem cells, and neurogenesis in the hippocampus [11,24,52,55]. Such chemotherapy-induced reductions in the neuro-genic and oligo-genic populations in the brain was linked with decreased BDNF and TrkB activity in the hippocampus [36,41]. The reduction of BDNF could also be a result of an inhibition of BDNF expression in the brain by cytokines.

In this study, we did not observe significant differential associations observed between cytokines and BDNF in patients without CRCI compared to patients who reported CRCI during the follow-up period. As the trajectory of impairment can be heterogenous among patients reporting CRCI, the associations between cytokines and BDNF were further evaluated in patients who experienced acute, persistent or delayed CRCI. Significant interaction was observed between persistent CRCI and IL-6, suggesting that there may be a divergence in the association between IL-6 and BDNF in this group of patients during chemotherapy. Previously, we have reported that a higher proportion of ESBC patients with persistent impairment had marked IL-6 increment compared to patients who had no impairment [53]. Although the interaction of CRCI trajectories and other cytokines did not reach statistical significance, the more divergent slopes or negative associations between many of the cytokines assessed and BDNF in patients with persistent CRCI may indicate a susceptibility factor for BDNF to the increase of cytokine levels. Patients with lower BDNF levels at the end of chemotherapy have been observed to be at higher risk of experiencing persistent CRCI [56]. In addition, the coefficient values as reflected in the divergent slopes for IL-2, IL-4, and TNF-α suggest possible implications of these cytokines in the pathophysiology of acute and persistent CRCI. Among patients with delayed CRCI, the inverse associations between cytokines and BDNF were weaker or closer to zero, compared to the other trajectories. The associations of cytokines and BDNF during chemotherapy may not be indicative in patients who developed cognitive impairment months after the completion of chemotherapy. Moreover, chemotherapy may not be the main contributing factor for cognitive impairment in these patients, as some may undergo radiation or endocrine therapy post chemotherapy.

There is a paucity of clinical studies investigating the association or interaction between circulating cytokines and BDNF, and available literature has reported contrasting outcomes in different clinical conditions. Similar to the findings from our cohort of ESBC patients, BDNF was predictive of cognitive performance, and a negative correlation was seen between IL-6 and BDNF in depressive cancer patients undergoing chemotherapy [25]. Conversely, Patas et al., found a positive correlation with BDNF for IL-6 but not TNF-α in patients with major depressive disorder [43]. They postulated that the correlation of peripheral BDNF and leucocyte count may represent the immunotropic origin of BDNF in their cohort. Zhang et al., observed poorer cognitive function in schizophrenic patients with lower BDNF levels and positive correlations between BDNF and both IL-2 and IL-8 levels [59]. The outcomes from these studies show that the relationship between cytokines and BDNF may vary depending on the diseases involved which differ in terms of etiology and signaling pathways implicated. These studies were also cross-sectional assessments, which may not reflect the dynamic changes and interaction of cytokines and BDNF over time.

Some cytokines may exhibit dual roles in the regulation of neurogenesis depending on the physiological condition, cell type involved, and signaling pathway activated [3,5,15,31,34,35,46]. For instance, increased TNF-α and IL-6 is archetypally associated with neuropsychological disorders precipitated by a pro-inflammatory environment, but can promote neuro-regeneration through activation of neurotrophic factors, in a physiologically unchallenged condition [3,35]. Likewise, elevation of IL-2 is implicated in the pathogenesis of neuropsychological conditions like depression and schizophrenia, although it is required for normal hippocampal function or BDNF signalling [5,15,49]. Microglial cells hold an integral position as the innate immune guardians in the brain, possessing the ability to shift into different phenotypic states depending on the interaction with other cells in the microenvironment. In a normal physiological state where cytokines balance is maintained, neuroglial pathways are inclined towards the maintenance of neuronal circuitry but during prolonged stress or chronic pathological conditions, pro-inflammatory and neurodegenerative pathways are activated [3,58].

In the event of a pro-inflammatory condition, which can be a consequence of chemotherapy, the microglia switches pathways to release IL-6, TNF-α and IL-1β, exacerbating inflammation, oxidative stress and neurodegeneration in the brain microenvironment [34]. In addition, IL-1β has been shown to inhibit neuronal BDNF expression in the presence of glial cells [46]. It is hypothesized that peripheral cytokines can have an indirect influence on cerebral BDNF levels by activation of microglial cells and in the current analysis, an inverse relationship between BDNF and pro-inflammatory cytokines IFN-γ, IL-1β, IL-2, IL-6, IL-8, GM-CSF and TNF-α may imply that [3]. Anthracyclines have been shown to elevate IL-1β and IFN-y levels and these cytokines are linked with increased microglial pro-inflammatory signaling leading to impaired cognitive function [2,21]. Preclinical studies demonstrated that chemotherapy treatment induced elevation of circulating and hippocampal TNF-α and IL-6 levels which impaired cognitive performance [51,52]. Interestingly, IL-4, an antiinflammatory cytokine was also inversely associated with BDNF levels. The increment in IL-4 levels throughout chemotherapy could be a compensatory response to the inflammatory cytokines and immune activation. Importantly, the equilibrium of Th1 and Th2 cytokines may be disrupted, which can lead to BDNF dysregulation and cognitive impairment [42]. Th1 cytokines such as IFN-γ, IL-2 and TNF-α are mostly pro-inflammatory, while Th2 cytokines like IL-4 and IL-10 are usually anti-inflammatory.

Clinical evidence on the associations of cytokines and cognitive function in cancer patients, including the types of cytokines involved, has been disparate [8]. This is likely due to the heterogeneity of cancer or treatment types, experimental design as well as the possible involvement of intermediary factors like BDNF. However, the underlying factors modifying the susceptibility of BDNF to the elevation of cytokine levels in CRCI patients remain unclear. Genetic variations can modify the risk and resilience to neuropsychological disorders including CRCI, and the BDNF Val66Met (rs6265) polymorphism has been reported to moderate the relationship between inflammation and depressive symptoms in women with ESBC [13]. In our cohort of patients with ESBC, there was no significant interaction of the rs6265 polymorphism with cytokines which would affect BDNF levels (data not shown). Other genetic variations or factors may be influencing the vulnerability of certain groups of patients with CRCI to the changes of BDNF levels, in association with cytokines.

As this was an explorative analysis, the associations of the peripheral biomarker levels were only indicative of a potential relationship or regulatory role between cytokines and BDNF, and did not necessarily equate to a direct causative effect. Additionally, the sample size after classification into the various CRCI trajectories (acute, persistent or delayed) may not be sufficiently powered to detect interaction effects of impairment status and cytokines. Subjective or self-reported measurement of CRCI was utilized in this analysis as our previous study has demonstrated a lack of significant association between BDNF and objective measures of CRCI [56]. Numerous studies have shown poor correlation between subjective and objective measures of CRCI [23]. A possible reason for this is that self-reported assessments may be more reflective of cognitive issues encountered in daily activities, which require the coordination of various cognitive skills, in comparison to structured objective neuropsychological tests conducted in controlled environments [22,23]. Objective measures may also lack the sensitivity to detect mild cognitive dysfunction experienced by cancer patients [18]. Subjective measures of cognitive function, on the other hand, may overlap with psychological distress perceived by a patient in view of cognitive impairment [23]. Hence, the CRCI community generally agrees that both objective and subjective measures are important for the assessment of CRCI [18].

Future work should include in vitro or in vivo functional studies to delineate the underlying mechanisms and draw conclusive inferences on the abilities of cytokines to modulate BDNF levels and the susceptibility to acute or persistent CRCI. Measurement of cytokines and BDNF levels after the completion of chemotherapy would also provide a clearer understanding of the relationship between these biomarkers especially in patients who develop delayed cognitive impairment. Nevertheless, the strength of this study lies in the longitudinal design as well as the wide array of cytokines investigated, which enabled the evaluation of cytokine levels in relation to BDNF and their trends during chemotherapy. Although the peripheral biomarker levels were only available throughout chemotherapy, the 12–24 months follow up period to assess cognitive function post-chemotherapy allowed for a comparison of the associations between cytokines and BDNF levels in different CRCI trajectories. Knowledge of the contributions of cytokines and BDNF dysregulation to CRCI could be leveraged for better management of this condition. Cytokines could represent valuable targets and biomarkers for the prevention of CRCI, as they can exert a direct or indirect impact, via BDNF, on cognitive function. For instance, physical exercise presents an attractive management strategy because it has been shown to improve cognitive function by restoring cytokine homeostasis and increasing BDNF levels [17,37,41].

In conclusion, cytokines were inversely associated with BDNF levels in ESBC patients with and without CRCI. The diverging trends for the associations between cytokines and BDNF were more distinctive when patients with CRCI were grouped by the different impairment trajectories. The differential associations between cytokines and BDNF throughout chemotherapy may be indicative of susceptibility for BDNF reduction to the increase of cytokines in patients experiencing acute or persistent CRCI. In particular, a significant interaction effect between IL-6 and persistent impairment was observed, which would impact on plasma BDNF levels. Findings from this study serve as a basis for future research to elucidate the specific interactions of cytokines and BDNF, along with their contributions to CRCI.

## Supplementary Material

Table S1 and S2

## Figures and Tables

**Table 1:** Demographic and clinical characteristics of study participants (N = 136).

Demographic characteristics		
Age in years, *mean* ± *SD*		52.0 ± 8.9
Ethnicity, *n* (%)	Chinese	116 (85.3)
	Malay	10 (7.4)
	Indian	5 (3.7)
	Others	5 (3.7)
Marital status, *n* (%)	Married	95 (69.9)
	Single	30 (22.1)
	Divorced	10 (7.4)
	Widowed	1 (0.7)
Highest education level, *n* (%)	Primary school	17 (12.5)
	Secondary school	67 (49.3)
	Pre-university	21 (15.4)
	Graduate/postgraduate	28 (20.6)
	Not reported	3 (2.2)
Occupational status at recruitment, *n* (%)	Employed	93 (68.4)
	Unemployed	43 (31.6)
Clinical characteristics		
Breast cancer stage, *n* (%)	I	15 (11.0)
	II	91 (66.9)
	III	30 (22.1)
ECOG status, *n* (%)	0	131 (96.3)
	1	5 (3.7)
Menopausal status, *n* (%)	Pre-menopausal	69 (50.7)
	Post-menopausal	67 (49.3)
Body mass index (BMI), *mean* ± *SD*		24.6 ± 4.5
BDNF genotype (rs6265), *n* (%)	GG (Val/Val)	36 (26.5)
	AG (Val/Met)	74 (54.4)
	AA (Met/Met)	26 (19.1)
Type of surgery, *n* (%)	Mastectomy	88 (64.7)
	Lumpectomy	48 (35.3)
Chemotherapy regimen, *n* (%)	Anthracycline-based	96 (70.6)
	Taxane-based	40 (29.4)
Local radiation post-chemotherapy, *n* (%)	Yes	97 (71.3)
	No	39 (28.7)
Hormonal therapy post-chemotherapy, *n* (%)	None	23 (16.9)
	Aromatase inhibitor	48 (35.3)
	Tamoxifen	65 (47.8)
Behavioral symptoms, *mean* ± *SD*	Baseline fatigue^a^	1.71 ± 1.99
	Baseline anxiety^b^	6.15 ± 5.43
	Baseline depression^c^	5.89 ± 7.05
CRCI trajectories, *n* (%)	No impairment	81 (59.6)
	Acute	15 (11.0)
	Persistent	21 (15.4)
	Delayed	19 (14.0)

a Total score of the BFI questionnaire is 10.

b Total score of the BAI questionnaire is 63.

c Total score of the BDI questionnaire is 63.

**Table 2:** Plasma cytokine and BDNF levels across time points (T1, T2 and T3).

Cytokines (pg/ml)	Median concentration (Interquartile range)			P value	Post-hoc^a^ P value		
	T1	T2	T3		T1 vs T2	T2 vs T3	T1 vs T3
IFN-γ	1.10 (0.04, 12.78)	1.21 (0.00, 11.53)	1.62 (0.02, 17.26)	**0.029**	0.918	**0.010**	0.053
IL-1β	0.08 (0.00, 0.31)	0.09 (0.00, 0.25)	0.12 (0.00, 0.38)	0.227	0.960	0.064	0.062
IL-2	0.00 (0.00, 0.38)	0.00 (0.00, 0.88)	0.00 (0.00, 0.63)	**0.032**	0.024	0.515	0.111
IL-4	0.01 (0.00, 0.29)	0.05 (0.00, 0.34)	0.18 (0.00, 0.53)	**<0.001**	0.042	**0.004**	**<0.001**
IL-6	0.41 (0.00, 1.04)	0.57 (0.00, 1.61)	0.63 (0.03, 1.87)	**0.002**	**<0.001**	0.038	**<0.001**
IL-8	3.52 (2.04, 4.86)	3.28 (2.10, 4.78)	3.60 (2.24, 5.28)	**0.007**	0.750	**0.002**	0.050
TNF-α	5.73 (1.61, 14.03)	5.46 (1.14, 17.52)	6.94 (1.48, 18.37)	**0.001**	0.518	**<0.001**	**0.004**
GM-CSF	0.00 (0.00, 0.10)	0.00 (0.00, 0.91)	0.00 (0.00, 4.35)	**<0.001**	0.027	0.072	**<0.001**
BDNF (ng/ml)	6.81 (4.71, 9.28)	5.75 (3.48, 8.04)	4.79 (3.26, 7.22)	**<0.001**	**<0.001**	0.018	**<0.001**

T1 : Before chemotherapy initiation, T2 : 6 weeks after chemotherapy initiation, T3 : At the end of chemotherapy.

a Wilcoxon sign-ranked test with Bonferroni adjustment, p < 0.017 is statistically significant.
