# Supplementary material for: Relationship between cytokines and brain-derived neurotrophic factor (BDNF) in trajectories of cancer-related cognitive impairment
Source: Cytokine. Author manuscript; Available in PMC 2021 Nov 12. (PMC8585614; doi:10.1016/j.cyto.2021.155556)
Supplement: Table S1 and S2 [file NIHMS1748102-supplement-Table_S1_and_S2.docx]

Table S1. Comparison of demographic and clinical characteristics for participants included and excluded in the final analysis

| Demographic characteristics | | Included in analysis  N = 136 | Excluded from analysis  N = 81 | | P value |
| --- | --- | --- | --- | --- | --- |
|  |  | Proportion (%) | | |  |
| Age in years*, mean ± SD* |  | 52.0 ± 8.9 | 52.3 ± 9.0 | 0.83 | |
| Ethnicity | Chinese | 85.3 | 71.6 | **0.04** | |
|  | Malay | 7.4 | 16.0 |  | |
|  | Indian | 3.7 | 9.9 |  | |
|  | Others | 3.7 | 2.5 |  | |
| Education level | Secondary school level and above | 86.6 | 85.5 | 0.65 | |
| BDNF SNP | GG (Val/Val)  AG (Val/Met)  AA (Met/Met) | 26.5  54.4  19.1 | 25.3  48.1  26.6 | 0.43 | |
| Clinical characteristics | |  |  |  | |
| Breast cancer stage | I & II | 77.9 | 83.7 | 0.10 | |
| ECOG status | 0 | 96.3 | 98.8 | 0.29 | |
| Menopausal status | Pre-menopausal | 50.7 | 42.0 | 0.21 | |
| Body mass index (BMI), *mean ± SD* | | 24.6 ± 4.5 | 24.5 ± 4.3 | 0.91 | |
| Type of surgery | Mastectomy | 64.7 | 62.5 | 0.74 | |
| Chemotherapy regimen | Anthracycline-based | 70.6 | 50.0 | **<0.01** | |
| Radiation therapy post-chemotherapy |  | 71.3 | 55.0 | **0.02** | |
| Hormonal therapy post-chemotherapy | None | 16.9 | 29.6 | 0.07 | |
|  | Aromatase inhibitor | 35.3 | 35.8 |  | |
|  | Tamoxifen | 47.8 | 34.6 |  | |
| Behavioral symptoms, *mean ± SD* | Baseline fatigue^a^ | 1.71 ± 1.99 | 1.54 ± 1.65 | 0.53 | |
|  | Baseline anxiety^b^ | 6.15 ± 5.43 | 7.80 ± 9.19 | 0.14 | |
|  | Baseline depression^c^ | 5.89 ± 7.05 | 6.67 ± 7.07 | 0.43 | |
|  |  |  |  |  | |

^b^Total score of the BFI questionnaire is 10

^c^Total score of the BAI questionnaire is 63

^d^Total score of the BDI questionnaire is 63

Table S2. Linear mixed model analysis investigating the associations between participant characteristics and BDNF levels

| Demographic characteristics | | Coefficient (95% CI) | P value |
| --- | --- | --- | --- |
| Age in years |  | -0.01 (-0.06 , 0.04) | 0.69 |
| Ethnicity | Chinese | ^d^ |  |
|  | Malay | 0.36 (-1.42 , 2.14) | 0.69 |
|  | Indian | 0.29 (-2.18 , 2.76) | 0.82 |
|  | Others | 0.20 (-2.27 , 2.67) | 0.87 |
| Education level | Primary school | ^d^ |  |
|  | Secondary school | 0.22 (-1.24 , 1.68) | 0.77 |
|  | Pre-university | 0.18 (-1.57 , 1.93) | 0.84 |
|  | Graduate/postgraduate | -0.58 (-2.23 , 1.06) | 0.49 |
| Clinical characteristics | | |  |
| Breast cancer stage | I | ^d^ |  |
|  | II | 0.59 (-0.92 , 2.09) | 0.44 |
|  | III | 0.53 (-1.18 , 2.23) | 0.55 |
| ECOG status | 0 | ^d^ |  |
|  | 1 | 0.52 (-1.95 , 2.98) | 0.68 |
| Menopausal status | Pre-menopausal | ^d^ |  |
|  | Post-menopausal | -0.47 (-1.40 , 0.45) | 0.31 |
| Body mass index (BMI) | | 0.07 (-0.03 , 0.18) | 0.15 |
| Type of surgery | Lumpectomy | ^d^ |  |
|  | Mastectomy | 0.16 (-0.81 , 1.13) | 0.74 |
| Chemotherapy regimen | Anthracycline-based | ^d^ |  |
|  | Taxane-based | 0.65 (-0.36 , 1.67) | 0.21 |
| BDNF SNP | Val/Val  Met carriers | ^d^  -0.40 (-1.45 , 0.65) | 0.45 |
| Behavioral symptoms | Fatigue^a^ | -0.09 (-0.27 , 0.09) | 0.31 |
|  | Anxiety^b^ | -0.02 (-0.08 , 0.03) | 0.46 |
|  | Depression^c^ | -0.01 (-0.07 , 0.05) | 0.73 |
|  |  |  |  |

^b^Fatigue measured using the BFI questionnaire

^c^Anxiety measured using the BAI questionnaire

^d^Depression measured using the BDI questionnaire

^d^Reference group

**A**

**B**

**C**

**D**

**E**

**F**

**G**

**H**

**I**

Figure S1. Standard curves for cytokines evaluated. LLOQ-ULOQ for the cytokines are (A) IFN-γ, 0.107-1822.276 pg/ml, (B) IL-1β, 0.024-378.967 pg/ml, (C) IL-2, 0.588-2494.390 pg/ml, (D) IL-4, 0.064-424.487 pg/ml, (E) IL-6, 0.075-1086.537 pg/ml, (F) IL-8, 0.456-1818.501 pg/ml, (G) IL-10, 0.706-2705.963 pg/ml, (H) GM-CSF, 0.218-897.805 pg/ml, (I) TNF-α, 0.469-6820.216 pg/ml

LLOQ : Lower limit of quantification

ULOQ : Upper limit of quantification
